# Clinical outcomes of a digitally supported approach for self-management of type 2 diabetes mellitus

**DOI:** 10.3389/fpubh.2023.1219661

**Published:** 2023-08-17

**Authors:** Vincenzo De Luca, Lutgarda Bozzetto, Clemente Giglio, Giovanni Tramontano, Giuseppina De Simone, Antonio Luciano, Luigi Lucibelli, Ada Maffettone, Michele Riccio, Geremia Romano, Ernesto Rossi, Carlos Juan Chiatti, Alexander Berler, Guido Iaccarino, Maddalena Illario, Giovanni Annuzzi

**Affiliations:** ^1^Dipartimento di Sanità Pubblica, Università degli Studi di Napoli Federico II, Naples, Italy; ^2^Dipartimento Assistenziale Integrato di Endocrinologia, Diabetologia, Andrologia e Nutrizione, Azienda Ospedaliera Universitaria Federico II, Naples, Italy; ^3^Dipartimento di Medicina Clinica e Chirurgia, Università degli Studi di Napoli Federico II, Naples, Italy; ^4^Unità Operativa Semplice Ricerca e Sviluppo, Azienda Ospedaliera Universitaria Federico II, Naples, Italy; ^5^Azienda Sanitaria Locale Napoli 3 Sud, Naples, Italy; ^6^Azienda Sanitaria Locale Benevento, Benevento, Italy; ^7^Azienda Ospedaliera di Rilievo Nazionale dei Colli, Naples, Italy; ^8^Azienda Sanitaria Locale Napoli 2 Nord, Naples, Italy; ^9^Tech4Care Srl, Ancona, Italy; ^10^Gnomon Informatics SA, Thessaloniki, Greece; ^11^Dipartimento di Scienze Biomediche Avanzate, Università degli Studi di Napoli Federico II, Naples, Italy

**Keywords:** digital health, mHealth, type 2 diabetes mellitus, self-management, patient empowerment, telemedicine, telehealth

## Abstract

**Background:**

Self-management of Type 2 diabetes mellitus (T2D) is challenging. Regular self-monitoring of blood glucose and healthy lifestyles are required to improve glycometabolic control, thus delaying diabetes complications, and reducing hospitalizations. Digital technologies can empower patients in their disease management promoting self-management and motivation to change behaviors. We report the results of an exploratory trial aimed at evaluating the metabolic outcomes of using digital solutions for T2D self-management developed in the ProEmpower project, a European Commission funded Pre-Commercial Procurement.

**Methods:**

Two digital solutions, DM4All and DiaWatch, which were codesigned with providers, patients, and caregivers, enabled the collection of clinical parameters by the patient using a smartphone integrated with the medical devices (glucometer, sphygmomanometer, scale, smart watch for heart rate monitoring and step counter). Data were automatically sent to the shared care plan allowing professionals to monitor adherence to treatment, set goals, and communicate more effectively with patients. At baseline and after an average follow-up of 8 months, glycosylated hemoglobin (HbA1c), body weight, blood pressure, and blood lipids were measured in 100 T2D patients using the ProEmpower solutions across different diabetes centers in Campania Region, age 45–79  years, both genders, and compared with a Control cohort of T2D patients (*n* = 100) with similar clinical characteristics and followed for a comparable period of observation in the same centers.

**Results:**

At baseline, the ProEmpower participants and the Control subjects were on average overweight, with a similar BMI in the two cohorts, and mean HbA1c was at acceptable levels (around 7.0%). After the 8 month exploratory trial, body weight, HbA1c, systolic and diastolic blood pressure, and plasma and LDL-cholesterol significantly decreased in the ProEmpower participants compared to baseline (*p* < 0.05 for all). The changes in systolic and diastolic blood pressure, and plasma and LDL-cholesterol were significantly different from those observed in the Control cohort (*p* < 0.05 for all).

**Conclusion:**

This pilot study showed positive effects on metabolic outcomes relevant to cardiovascular risk in T2D of adopting digital telemedicine self-monitoring solutions based on automation of measurements and coaching on healthy lifestyles promotion.

## 1. Introduction

Type 2 Diabetes Mellitus (T2D) is a metabolic disease characterized by many concomitant conditions, severe complications, and spreading to all age groups (1). T2D has taken on the characteristics of a real health emergency due to its high prevalence. There are approximately 537 million people suffering from diabetes worldwide and this number is set to increase to 783 million by 2045 (2). Campania is one of the Italian regions with the highest diabetes prevalence rates. In 2020, there were approximately 427,000 patients with diabetes in Campania, equal to 7.6% of the total population, significantly higher than the average figure for Italy (5.9%). Campania shows high levels of diabetes prevalence for both females and males (respectively 7.4 and 7.6%) and among older adults the shares stand at 27.5% in females and 27, 1% in males. Campania has the highest diabetes mortality rate in Italy. 5 deaths per 10,000 for males and 4 per 10,000 for females occur from causes attributable to diabetes (3).

Diabetes requires numerous decisions to be made in patients’ everyday life regarding drug therapy, glycemic control, nutrition, physical activity, family, work and social relationships. Diabetes self-management is the process of enabling patients to acquire knowledge, practical skills, and behavioral skills to apply in day-to-day management of their disease (4). Self-management of diabetes is challenging, as it requires patient’s motivation and commitment to prevent complications of the disease. Specifically, regular self-monitoring of blood glucose and healthy lifestyles, including physical exercise, diet, and medication adherence, is required to improve glycemic control, thus delaying diabetes complications, and reducing hospitalizations (5). One of the main obstacles to the optimal management of T2D is the poor capacity of service delivery systems to share clinical information, which either duplicates services or compartmentalizes them (6, 7).

The Chronic Care Model (CCM) states that better health outcomes require an informed and active patient and a proactive team. Clinical information systems and decision-support tools play a key role in enabling the patient and the practitioner to interact effectively while improving health outcomes (8). The COVID-19 pandemic accelerated the adoption of digital solutions for health. During pandemic, hospitals and healthcare organizations relied on innovative approaches and technologies to support patients, unable to visit healthcare facilities due to restrictions imposed by governments to prevent new infections, (9). The development of digital technologies increased the implementation of innovative ways of communication and organizational approaches, expanding from health to entertainment, business, education, to strengthen the management and prevention of chronic diseases, such as T2D self-care (10).

Telemedicine has been shown to be feasible and effective for diabetes health care delivery and disease self-management, but they require adaptation to the institution, clinician, and patient population (11). A personalized and integrated approach by healthcare professionals and health information technologies (IT) would support patients to be motivated to cope effectively with diabetes (10). Specifically, shared decision-making tools can empower patients in their disease management decisions, just as online platforms support patients in communicating with their peers and health professionals, promoting self-management and health literacy (12). Mobile apps for coaching interventions improve patients’ motivation to change their behavior, promoting goals, and reducing health risks (13). Consistency with the experience of care, held only by the patient, and the integration into the work routines of care processes and daily lives of professionals makes investment in new digital solutions effective (14).

“Procuring innovative ICT for patient empowerment and self-management of type 2 diabetes mellitus” (ProEmpower) is an European Commission funded Pre-Commercial Procurement (15–17) for research and development services aimed at developing innovative digital solutions to improve T2D integrated care, patient empowerment and self-management. Through a patient-centered approach, four public procurers from Turkey, Portugal, Spain and Italy respectively, defined an innovative diabetes management process, supported by fully integrated IT solutions for diabetes self-management and remote patient monitoring (18, 19). The ProEmpower project led to the co-design of two solutions, DM4All and DiaWatch, aimed at promoting self-care and continuous monitoring through integrated IT systems comprising web and mobile interfaces and smart medical devices.

Aim of this study was to assess how the ProEmpower solutions affected key metabolic parameters relevant in the management and prognosis of T2D patients. Data were obtained during an exploratory trial aimed at evaluating the outcomes of using the ProEmpower solutions in a cohort of patients from diabetes centres across Campania Region, participating in the project.

## 2. Materials and methods

### 2.1. Study design and setting

The study aimed to analyze glycosylated hemoglobin, body weight, blood pressure, and blood lipids in the participants who actively used the ProEmpower solutions for self-management of T2D. Data were collected at baseline and after an average follow-up of 8 months and were compared with the data of a cohort of patients with T2D, with similar clinical characteristics and followed for a comparable period of observation. The diabetes center at the Federico II University Hospital (Azienda Ospedaliera Universitaria Federico II) was the coordinator of the exploratory trial in Campania, in collaboration with seven health centers across the region willing to join. Eligible, consented patients were randomized for using either DM4All or DiaWatch. Randomization was performed at level of healthcare center. Each center used a single solution, adopting the same protocol for enrolment and monitoring patients.

The ProEmpower solutions enable the collection of clinical parameters by the patient, using a smartphone integrated with the medical devices. The data collected by the integrated devices (glucometer, sphygmomanometer, scale, smart watch for heart rate monitoring and step counter) were automatically sent to the shared care plan. The shared care plan, accessed through the patient and the professional profiles, includes information on lifestyle, treatment plan, and disease-related data. The interface for professionals allows them to monitor adherence to treatment, set goals, and communicate more effectively with patients. The solutions automatically send suggestions to patients when they deviate from treatment targets.

This research follows the recommendations of the UK’s Medical Research Council (MRC) to first use modelling and exploratory studies before targeting randomized controlled trials (RCTs) and long-term implementation. This allows for the continuous development and iterative improvement of new technologies in a continuum of growing evidence (20). Therefore, the approach fits the nature of pre-commercial procurement projects. The study is performed in accordance with the article 89 of the General Data Protection and Regulation, which allows the processing of personal data for archiving purposes in the public interest, scientific or historical research purposes or statistical purposes, provided that technical and organizational measures are in place in order to ensure the principle of data minimization (21).

### 2.2. Clinical centres participating in the pilot study

**Figure 1:**
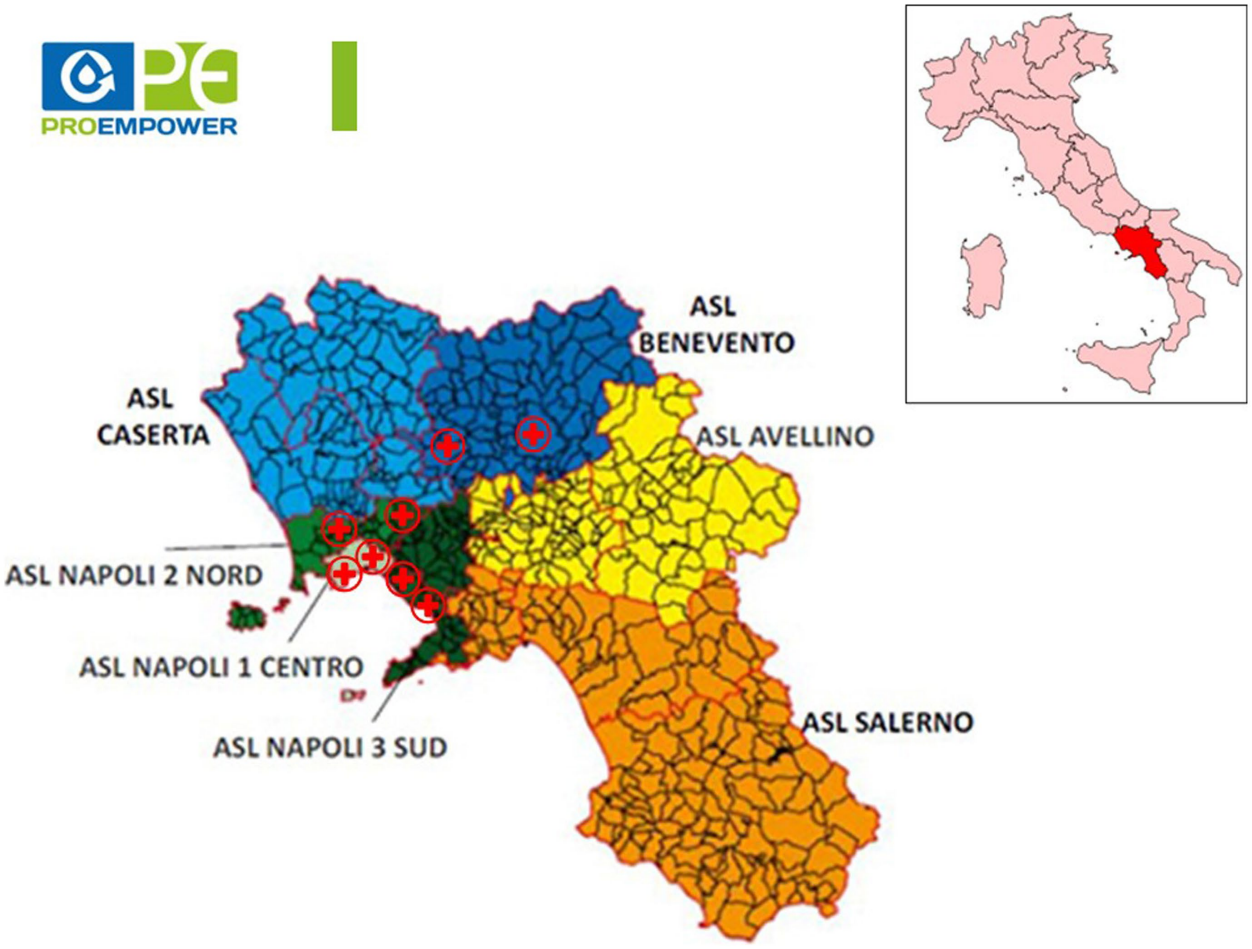
Location of participating centres. The figure shows the location of the centres participating in the ProEmpower project on the map representing the Local Health Agencies of the Campania region (Italy).

The study included the following centers: Diabetes Unit, “Federico II” University Hospital; “Benevento 2” and “Montesarchio” Health Districts, “Benevento” Local Health Agency; “Casalnuovo di Napoli” Health District, “Napoli 2 Nord” Local Health Agency; “Marano di Napoli” Health District, “Napoli 2 Nord” Local Health Agency; “San Giorgio a Cremano” Health District, “Napoli 3 Sud” Local Health Agency; “Torre Annunziata” Health District, “Napoli 3 Sud” Local Health Agency; Metabolic Unit, “Azienda dei Colli” National Relevance Hospital, Naples (Figure 1).

### 2.3. ProEmpower participants

One hundred T2D patients (50 for each solution) were enrolled. The exploratory trial was performed un-blinded, and all participants knew they were part of an intervention. Similarly, healthcare professionals knew that they were treating a ProEmpower patient. Subjects with the following inclusion criteria were eligible to participate to the project: age between 45 and 79 years, both genders, diagnosis of type 2 diabetes mellitus (from recently diagnosed to long standing diabetes); ability and willingness to provide written informed consent. Subjects were excluded if pregnant or being on renal replacement therapy (hemodialysis, peritoneal dialysis, or transplantation), or having a history of active malignancy within the last 5 years, chronic viral hepatitis or other serious illnesses.

### 2.4. Control cohort

Data from 100 patients with T2D were consecutively collected in the same diabetes centers participating in the pilot study. Data were taken at baseline and after 6–8 months, corresponding to a similar duration of follow-up as for the ProEmpower participants. The baseline clinical characteristics of the T2D control group and the ProEmpower participants in the pilot study are reported in Table 1.

**Table 1:** Baseline clinical characteristics.

	ProEmpower participants	Control subjects	*p* value
Sex (M/F)	83/17	70/30	0.020
Age (years)	61.1 ± 9.4	66.5 ± 9.0	<0.001
Body weight (kg)	86.4 ± 14.3	80.9 ± 16.1	0.015
Height (cm)	171 ± 8	167 ± 7	<0.001
Body mass index (kg/m^2^)	29.6 ± 5.0	29.0 ± 5.6	0.460
HbA1c (%)	6.99 ± 0.95	7.04 ± 0.93	0.753
M ± SD			

The table reports the baseline clinical characteristics of the T2D ProEmpower participants in the pilot study and the control T2D subjects.

### 2.5. Measurements

Before and after the pilot study the following outcomes were collected and analyzed: body weight, HbA1c, blood pressure, and blood lipids. The study period coincided with the start of COVID pandemic and the lockdown restrictions (September 2019–July 2020). This made difficult for both ProEmpower and Control participants to make the final visit at the end of the observation period. Therefore, clinical and biochemical data corresponding to the end of the pilot study were not available for many participants and the obtainable before-after comparisons in the ProEmpower cohort ranged from around 90% for body weight, HbA1c, and blood pressure to around 40% for blood lipids.

### 2.6. Statistical analysis

Data are expressed as mean ± SD unless otherwise stated. Within the group, baseline to end of observation differences were assessed by paired sample *t*-test. The differences between the changes in the ProEmpower cohort and the Control cohort were evaluated by *t*-test for independent samples. A value of *p*<0.05 was considered significant. The statistical analysis was performed according to standard methods using the SPSS software V.28 (SPSS/PC).

## 3. Results

As shown in Table 1, in the ProEmpower cohort men were more represented and age was significantly lower than in the Control cohort, with no differences in BMI and glycated hemoglobin between the two cohorts. Data taken at baseline and after 6–8 months are summarized in Table 2.

**Table 2:** Clinical outcomes.

	ProEmpower participants				Control subjects				Difference between two cohorts
	*n*	Baseline	8 Months	*p* value	*n*	Baseline	8 Months	*p* value	*p* value
HbA1c (%)	84	6.99 ± 0.95	6.76 ± 0.87	0.019	100	7.04 ± 0.93	6.90 ± 0.87	0.047	0.555
Body weight (Kg)	89	86.0 ± 13.8	83.7 ± 16.0	0.019	94	81.2 ± 16.0	79.9 ± 17.9	0.072	0.385
Systolic blood pressure (mmHg)	92	135.6 ± 18.0	126.1 ± 14.5	<0.001	39	131.4 ± 16.9	132.7 ± 15.0	0.303	<0.001
Diastolic blood pressure (mmHg)	92	80.5 ± 9.8	76.2 ± 8.9	<0.001	39	78.2 ± 7.7	79.9 ± 7.6	0.102	<0.001
Plasma cholesterol (mg/dL)	46	179.9 ± 49.3	158.1 ± 37.9	<0.001	59	166.4 ± 38.1	164.5 ± 35.4	0.654	0.003
Plasma triglycerides (mg/dL)	45	139.9 ± 81.3	133.4 ± 81.1	0.544	55	140.1 ± 56.8	147.0 ± 54.9	0.250	0.252
LDL cholesterol (mg/dL)	39	109.1 ± 43.2	86.0 ± 32.1	<0.001	52	91.1 ± 31.7	88.4 ± 31.9	0.516	0.002
HDL cholesterol (mg/dL)	48	43.0 ± 9.9	44.4 ± 10.0	0.186	59	46.2 ± 12.6	45.2 ± 12.4	0.310	0.095
M ± SD									

The table reports the body weight, glucose control, blood pressure, and blood lipids outcomes in ProEmpower participants and Control subjects at baseline and after the 8-month observation period.

### 3.1. Blood glucose control

**Figure 2:**
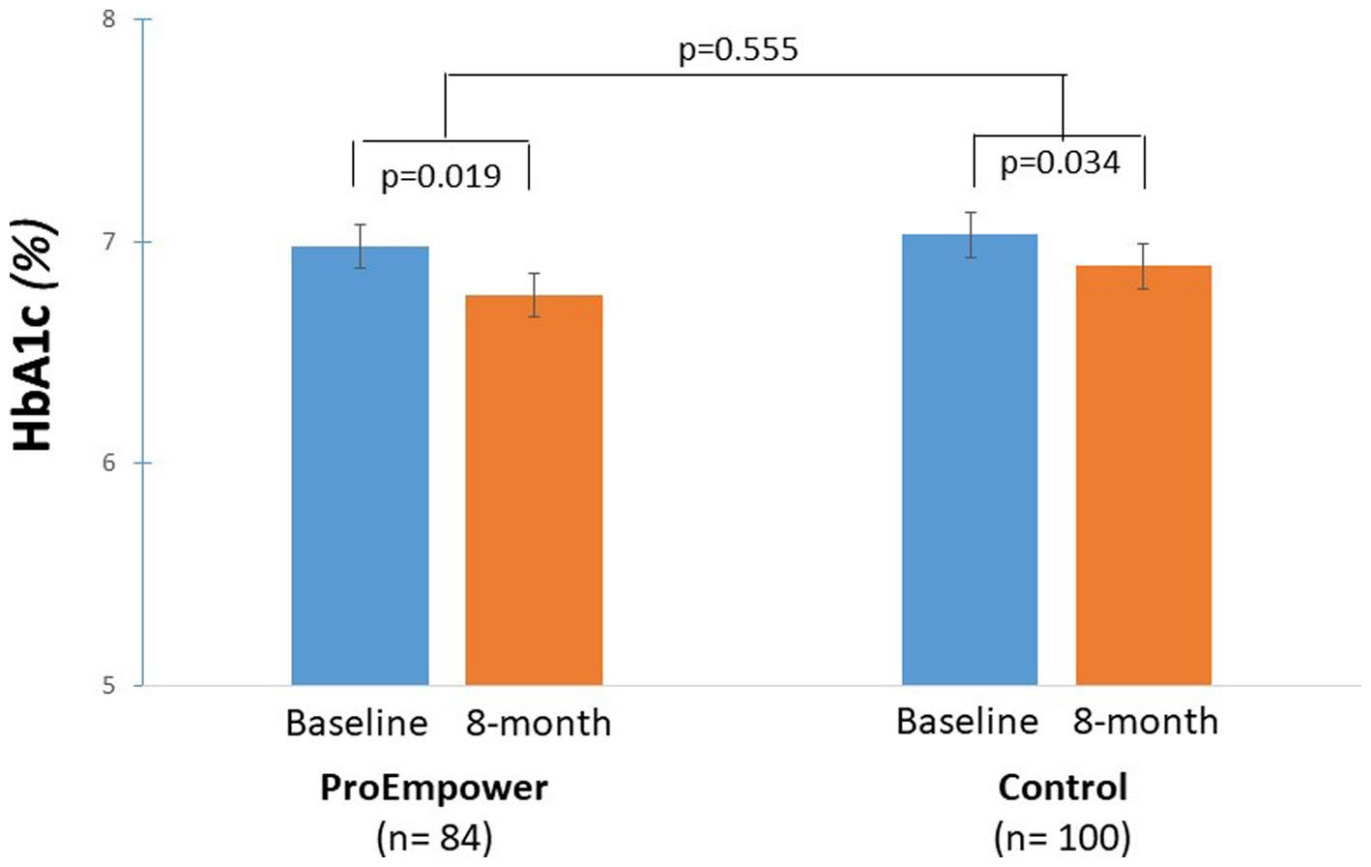
Blood glucose control outcomes. The figure shows the level of HbA1c before and after the 8-month observation period in the T2D patients participating in the ProEmpower Project and in the usual-care T2D control cohort. M ± SE.

At baseline, mean HbA1c was at acceptable levels (around 7.0%) both in the ProEmpower and the Control cohorts (Table 1). As shown in the Figure 2, after the 8 month exploratory trial, compared to baseline, HbA1c significantly decreased in the ProEmpower participants (6.99 ± 0.95 vs. 6.76 ± 0.87%, *p* = 0.019) and in the Control cohort (7.04 ± 0.93 vs. 6.90 ± 0.87%, *p* = 0.047), with no significant difference between the changes in the two cohorts (*p* = 0.555).

### 3.2. Body weight

**Figure 3:**
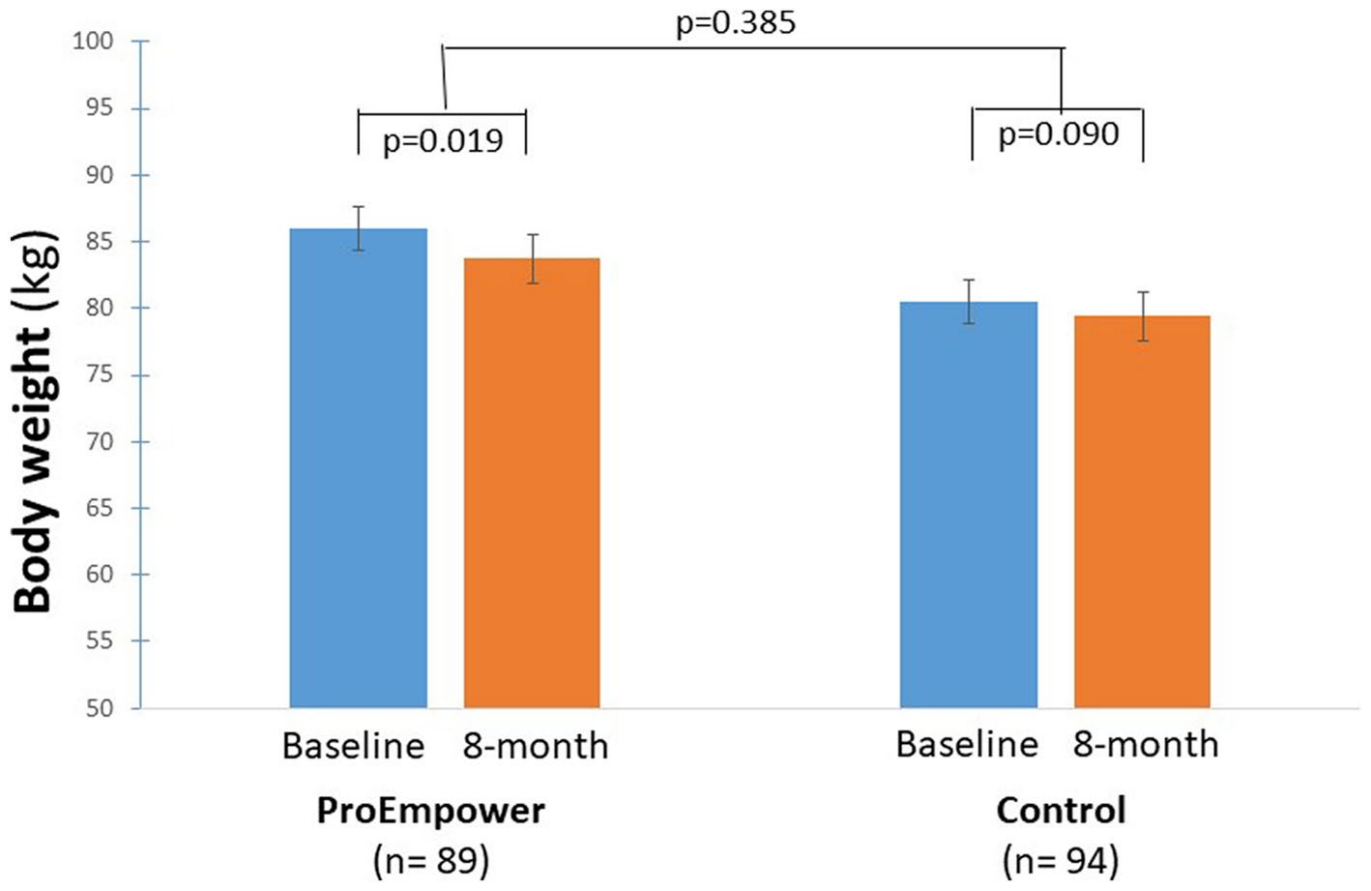
Body weight control outcomes. The figure shows the body weight outcomes before and after the 8-month observation period in the T2D patients participating in the ProEmpower Project and in the usual-care T2D control cohort. M ± SE.

The ProEmpower participants and the Control subjects were on average overweight, with a similar BMI in the two cohorts (Table 1). As shown in Figure 3, after the exploratory trial, compared to baseline, body weight decreased significantly in the ProEmpower participants (86.0 ± 13.8 vs. 83.7 ± 16.0 kg, *p* = 0.019) and not significantly in the Control cohort (81.2 ± 16.0 vs. 79.9 ± 17.9 kg, *p* = 0.072), with no significant difference between the changes in the two cohorts (*p* = 0.385).

### 3.3. Blood pressure

**Figure 4:**
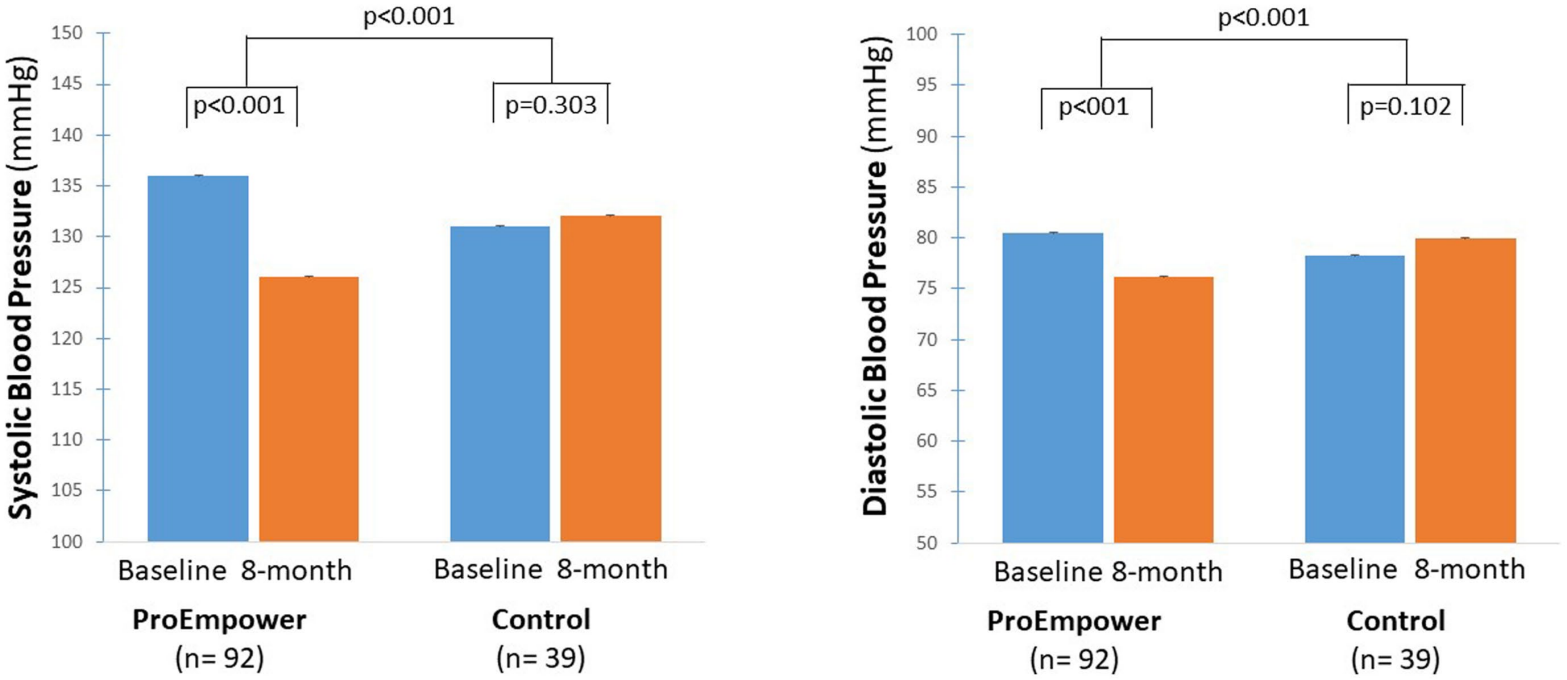
Blood pressure outcomes. The figure shows the Systolic and diastolic blood pressure outcomes before and after the 8-month observation period in the T2D patients participating in the ProEmpower Project and in the usual-care T2D control cohort. M ± SE.

As highlighted by Figure 4, after the exploratory trial, compared to baseline, systolic blood pressure decreased significantly in the ProEmpower participants (135.6 ± 18.0 vs. 126.1 ± 14.5 mmHg, *p* < 0.001), while it did not change in the Control cohort (131.4 ± 16.9 vs. 132.7 ± 15.0 mmHg, *p* = 0.303), with a significant difference between the changes in the two cohorts (*p* < 0.001).

As well as, diastolic blood pressure decreased significantly in the ProEmpower participants (80.5 ± 9.8 vs. 76.2 ± 8.9 mmHg, *p* < 0.001), while it did not change in the Control cohort (78.2 ± 7.7 vs. 79.9 ± 7.6 mmHg, *p* = 0.102), with a significant difference between the changes in the two cohorts (*p* < 0.001) (Figure 4).

### 3.4. Blood lipids

**Figure 5:**
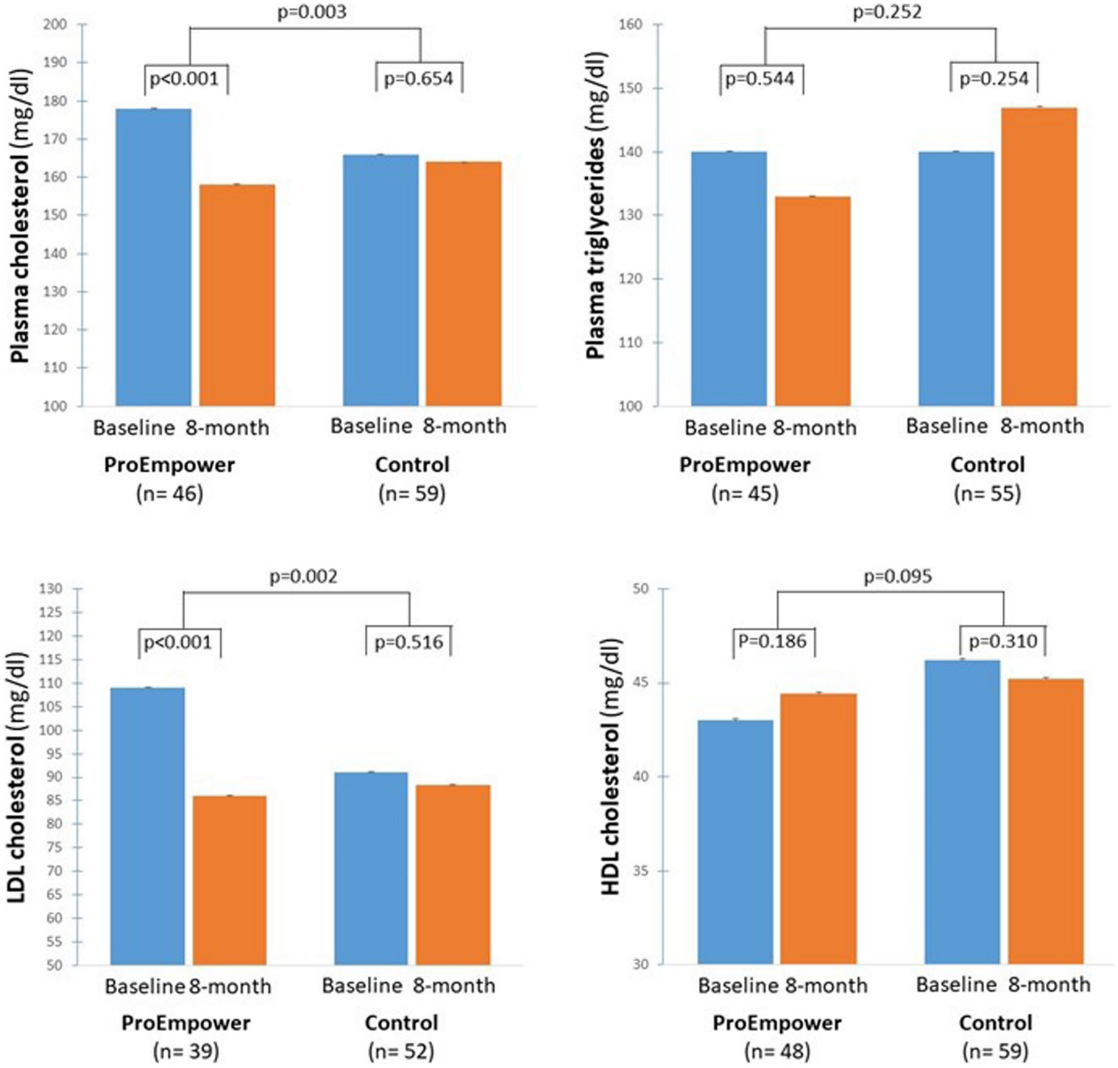
Blood lipids outcomes. The figure shows the plasma cholesterol, plasma triglycerides, LDL and HDL Cholesterol concentrations before and after the 8-month observation period in the T2D patients participating in the ProEmpower Project and in the usual-care T2D control cohort. M ± SE.

As shown in Figure 5, plasma cholesterol and LDL-cholesterol decreased significantly in the ProEmpower participants (179.9 ± 49.3 vs. 158.1 ± 37.9 mg/dL, *p* < 0.001, and 109.1 ± 43.2 vs. 86.0 ± 32.1 mg/dL, *p* < 0.001, respectively), while did not change in the Control cohort (166.4 ± 38.1 vs. 164.5 ± 35.4 mg/dL, *p* = 0.654, and 91.1 ± 31.7 vs. 88.4 ± 31.9 mg/dL, *p* = 0.516, respectively), with a significant difference between the changes in the two cohorts (*p* = 0.003 for plasma cholesterol, *p* = 0.002 for LDL-cholesterol).

Whereas, plasma triglycerides and HDL cholesterol did not change significantly in the ProEmpower participants (139.9 ± 81.3 vs. 133.4 ± 81.1 mg/dL, *p* = 0.544, and 43.0 ± 9.9 vs. 44.4 ± 10.0 mg/dL, *p* = 0.186, respectively), and in the Control cohort (140.1 ± 56.8 vs. 147.0 ± 54.9 mg/dL, *p* = 0.250, 46.2 ± 12.6 vs. 45.2 ± 12.4 mg/dL, *p* = 0.310, respectively), with no significant difference between the changes in the two cohorts (*p* = 0.252 for triglycerides, *p* = 0.095 for HDL-cholesterol) (Figure 5).

## 4. Discussion

Digital solutions enable better self-management of T2D, increasing adherence to medication and monitoring of blood glucose levels, decreasing the risk of complications and adverse events (22, 23). The present study aimed to analyze the changes in metabolic parameters among the participants who used two digital telemedicine solutions for self-management of T2D developed in the ProEmpower Pre-Commercial Procurement project.

### 4.1. Clinical outcomes

The results show that several metabolic outcomes, relevant to cardiovascular risk in people with T2D, were significantly improved in the ProEmpower participants after the 8 month observation period, namely body weight, glucose control, blood pressure, and plasma lipids. The changes in blood pressure and plasma lipids were significantly different from those observed over a similar period in a cohort of T2D patients with similar characteristics (Figures 4, 5). Several reasons may explain the changes in metabolic outcomes detected at the end of the observation period. A role could have been played by lifestyle modifications, in terms of dietary and physical activity changes, that were favored by the digital approach through the monitoring of dietary habits (daily food records) and physical activity (step number, heart rate), and the coaching on healthy lifestyle. This is in line with the reduction in body weight observed during the observation (Figure 3), in spite of the restrictions for the COVID-19 pandemic, likely fostered by the frequent use of the digital scale automatically connected to the shared care plan. In addition to the effects of lifestyle changes, the clinically significant improvements in systolic and diastolic blood pressure, and plasma and LDL cholesterol observed with the use of the digital tools could be due to increased adherence to drug therapy (antihypertensive agents, statins) as a result of the more intensive monitoring of blood pressure and the participation in the trial (24, 25). It must be mentioned that lipid profile has been previously reported to tendentially improve after COVID-19 lockdown in people with T2D (26).

Although blood glucose control at baseline was on average good in the ProEmpower and the control group, it significantly improved over the 8 month observation period in both groups (Figure 2). The improvement in glucose control was in line with that reported during and immediately after COVID-19 pandemic lockdown in people with type 1 diabetes or, less consistently, with T2D, particularly patients using insulin (26–28). In this study, the improvement was observed also in the control group likely reflecting more time for home glucose monitoring and therapy adjustments.

### 4.2. Impact of digital solutions on TD2 self-management

The digital solutions used in this study were codesigned with providers, patients, and caregivers, with the aim of empowering patients to promote lifestyle changes. Since the automation and pervasiveness of the digital solutions in implementing T2D management tasks is the most relevant factor influencing compliance, but also data correctness and certainty, maximal attention was given to full automation of data collection and transmission. One concern when using new technologies is the possible barrier of poor digitalization often related to advanced age. The mean age of the participants was over 60 years; however, the results of this study in terms of metabolic outcomes were not influenced by the age of the participants, confirming the potential use of digital approaches in this age population.

The participants who used the ProEmpower solutions gave a favorable opinion on using them, reporting a positive experience during the test phase and an increased ability to manage diabetes (29). In an increasing burden scenario for health systems, telemedicine solutions can provide optimal healthcare services for the management of T2D, with reduced costs and saving time for providers and patients.

### 4.3. Limitations

In interpreting the results, some limitations of the study must be considered. First, it was an exploratory trial for digital products being developed, and, therefore, there was no randomization of the participants. To approach this shortcoming, we compared the results in the ProEmpower group with those observed in a comparable group of patients followed by the same diabetes centres. Another limitation is that this pilot study started just before the lockdown for COVID-19 pandemic, and, therefore, the results might have been different if the study had been done in a non-pandemic time. The restrictive measures for the pandemic affected the completeness of the data because they did not allow all enrolled patients to complete follow-up visits and obtain the scheduled measurement. On the other hand, the results show that the ProEmpower telehealth approach made it possible to continue caring for these chronic patients despite the significant restrictive measures.

## 5. Conclusion

The present study aimed to analyze glycosylated hemoglobin, body weight, blood pressure, and blood lipids in the participants who actively used the ProEmpower solutions for self-management of T2D. Data were collected at baseline and after an average follow-up of 8 months and were compared with the data of a cohort of patients with T2D, with similar clinical characteristics and followed for a comparable period of observation. The study showed the positive effects on metabolic outcomes in T2D of adopting digital telemedicine self-monitoring solutions based on automation of clinical measurements and coaching on healthy lifestyles promotion. Our results are encouraging and suggest performing randomized controlled trials to confirm the effects of the specific features of the utilized solutions on health outcomes.

## Data availability statement

The raw data supporting the conclusions of this article will be made available by the authors, without undue reservation.

## Author contributions

VL, MI, and GA: conceptualization. VL, GA, LB, CG, GT, GS, AL, LL, AM, MR, GR, ER, CC, and AB: methodology. VL, GA, and MI: writing—original draft preparation. GI, MI, and GA: writing—review and editing. All authors contributed to the article and approved the submitted version.

## Funding

The present research has received funding from the European Commission’s Horizon 2020 research and innovation programme under the Grant Agreement No. 727409.

## Conflict of interest

CC is employed by Tech4Care Srl. AB is employed by Gnomon Informatics SA.

The remaining authors declare that the research was conducted in the absence of any commercial or financial relationships that could be construed as a potential conflict of interest.

## Publisher’s note

All claims expressed in this article are solely those of the authors and do not necessarily represent those of their affiliated organizations, or those of the publisher, the editors and the reviewers. Any product that may be evaluated in this article, or claim that may be made by its manufacturer, is not guaranteed or endorsed by the publisher.
